# Significant association between functional microRNA polymorphisms and coronary heart disease susceptibility: a comprehensive meta-analysis involving 16484 subjects

**DOI:** 10.18632/oncotarget.14249

**Published:** 2016-12-27

**Authors:** Xu Liu, Lianghao You, Ruizhi Zhou, Jian Zhang

**Affiliations:** ^1^ Department of Neurology, First Affiliated Hospital of China Medical University, Liaoning, Shenyang 110001, China; ^2^ Department of Cell Biology, Key Laboratory of Cell Biology, Ministry of Public Health, China, Key Laboratory of Medical Cell Biology, Ministry of Education, China Medical University, Shenyang 110122, China

**Keywords:** microRNA, polymorphism, coronary heart disease, meta-analysis, Pathology Section

## Abstract

Molecular epidemiological studies suggest that microRNA polymorphisms may be associated with an increased risk of coronary heart disease (CHD). However, the results of these studies were inconsistent and inconclusive. To derive a more precise evaluation, we performed a meta-analysis focused on the associations between microRNA polymorphisms and CHD risk. PubMed, Embase, CNKI and Wanfang databases were searched. Odds ratios (ORs) with 95% confidence intervals (CIs) were applied to assess the association between microRNA-146a rs2910164, microRNA-196a2 rs11614913, microRNA-499 rs3746444 and microRNA-149 rs71428439 polymorphisms and CHD susceptibility. Heterogeneity, publication bias and sensitivity analysis were conducted to measure the robustness of our findings. A total of thirteen related studies involving 8,120 patients and 8,364 controls were analyzed. Significant associations between microRNA-146a rs2910164 polymorphism and CHD risk were observed in the total population, as well as in subgroup analysis. For microRNA-196a2 rs11614913 and microRNA-499 rs3746444, similarly increased risks were also found. In addition, no significant association was detected between microRNA-149 rs71428439 polymorphism and CHD risk. In conclusion, our meta-analyses suggest that microRNA polymorphisms may be associated with increased risk of CHD development.

## INTRODUCTION

Coronary heart disease (CHD) has become a main cause of morbidity and mortality worldwide [1]. In 2010, approximately 7,000,000 deaths were reported globally, and in which CHD took up the largest proportion of death causes and years of life lost [2]. Traditional factors, such as hypertension, diabetes and smoking have been proven to contribute to the occurrence and progression of CHD [3–5]. However, more existed risk factors leading to CHD susceptibility need to be explored. Till now, increasing molecular epidemiological studies have revealed the important role of genetic factors in CHD, and the genetic predisposition is attracting more and more attention [6, 7].

MicroRNAs (miRNAs) are small single-stranded non-coding RNA molecules which function in the post-transcriptional regulation of gene expression [8]. Emerging evidence has indicated that the functions of miRNAs appear to be in a variety of fundamental biological processes, involving proliferation, differentiation and stress resistance [9–11]. In addition, recent studies have shown that miRNAs take part in the regulation of glucose and lipid metabolism, the proliferation of smooth muscle cells and vascular inflammation, which play important roles in the pathogenesis of CHD [12–16].

By affecting the miRNA maturation and the binding to target mRNAs, single nucleotide polymorphisms (SNPs) located in pre-microRNA (pre-miR) genes may alter the expression levels of a large number of target genes and cause the complex functional consequences [17]. Therefore, functional SNPs in miRNA genes may affect disease susceptibility. Previous studies have confirmed that four common miRNA polymorphisms (rs2910164 G>C in miR-146a, rs11614913 T>C in miR-196a2, rs3746444 A>G in miR-499 and rs71428439 A>G in miR-149) were associated with several diseases, including various cancers and autoimmune diseases [18–21]. Recently, these four SNPs were under investigation to uncover the possible genetic predisposing to CHD, but the results were inconsistent. Therefore, we conducted a meta-analysis involving all related publications to assess the association between microRNA polymorphisms and CHD risk.

## RESULTS

### Characteristics of studies

**Figure 1:**
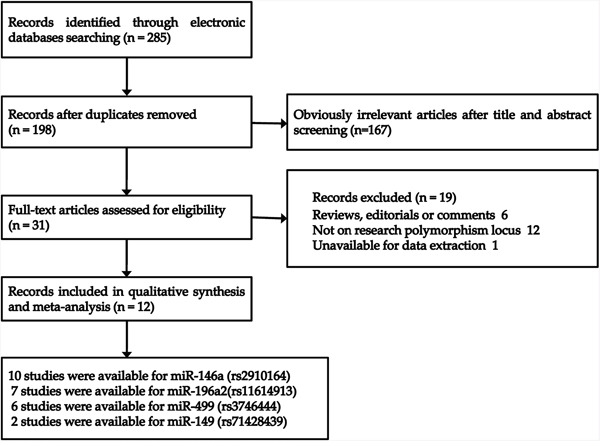
Flow diagram of the study selection process

In total, 285 relevant publications were retrieved according to the search strategy. Firstly, we excluded 254 articles after title reviewing and duplicate screening. Then, 19 studies including 6 reviews, 12 studies not for focus polymorphisms, and 1 study without available information [22] were excluded. Finally, 12 eligible articles (13 studies) published from 2012 to 2016 were selected in the meta-analysis, including ten studies on microRNA-146a rs2910164 G>C [23–32], seven studies on microRNA-196a2 rs11614913 T>C [23, 26, 27, 29, 31, 33], six publications on microRNA-499 rs3746444 A>G [23, 25, 26, 29, 31, 33], and two studies on microRNA-149 rs71428439 A>G [29, 34], respectively. The process of study selection was shown in Figure 1. Among the retrieved articles, nine articles [23, 24, 26–30, 33, 34] were written in English and three [25, 31, 32] in Chinese. Moreover, two of the studies involved Caucasians [24, 30], and eleven of them were conducted for Asians. The distribution of genotype was consistent with HWE in all studies but one study for microRNA-146a rs2910164 [32] and two for microRNA-499 rs3746444 polymorphism [25, 31]. Detailed characteristics of included studies were shown in Table 1.

**Table 1:** Characteristics of case-control studies on microRNA polymorphisms and CHD risk included in the meta-analysis

First author	Year	Country/Region	Ethnicity	Source of controls	Case	Control	Genotype distribution						Genotyping methods	Age and sex matched	*P for HWEa*
							Case			Control					
microRNA-146a rs2910164 G>C							CC	GC	GG	CC	GC	GG			
Sung JH	2016	Korea	Asian	Hospital	522	535	203	242	77	202	260	73	PCR-RFLP	matched	0.460
Bastami M	2016	Iran	Caucasian	NA	300	300	34	155	111	22	128	150	Taqman	matched	0.454
Huang SL	2015	China	Asian	Hospital	722	721	266	308	143	237	348	132	Taqman	matched	0.830
Xiong XD	2014	China	Asian	Hospital	295	283	113	141	41	97	125	61	PCR-RFLP	unmatched	0.086
Prithiksha R	2014	South Africa	Asian	NA	106	100	13	43	50	9	46	45	PCR-RFLP	matched	0.569
Chen CR	2014	China	Asian	Hospital	919	889	187	463	269	153	435	301	PCR-LDR	unmatched	0.846
Hamann L	2014	Germany	Caucasian	Population	206	200	12	74	120	10	73	117	PCR-HRM	unmatched	0.748
Chen L	2013	China	Asian	Hospital	658	658	172	305	181	134	330	194	Taqman	matched	0.769
Yang Y-a	2012	China	Asian	Population	853	948	272	392	165	271	457	189	Taqman	matched	0.885
Li L	2012	China	Asian	Hospital	415	1010	149	184	82	345	455	210	PCR-RFLP	unmatched	0.009
microRNA-196a2 rs11614913 T>C							CC	TC	TT	CC	TC	TT			
Sung JH	2016	Korea	Korean	Hospital	522	535	107	236	179	108	274	153	PCR-RFLP	matched	0.465
Huang SL	2015	China	Asian	Hospital	722	721	147	381	190	156	360	204	Taqman	matched	0.905
Xiong XD	2014	China	Asian	Hospital	295	283	78	131	86	68	132	83	PCR-RFLP	unmatched	0.278
Chen CR	2014	China	Asian	Hospital	919	889	157	450	312	161	406	322	PCR-LDR	unmatched	0.097
Zhi H	2012	China	Asian	Hospital	916	584	155	470	291	98	278	208	PCR-RFLP	matched	0.755
Yang Y-a	2012	China	Asian	Population	853	948	163	463	202	217	463	241	Taqman	matched	0.853
Yang Y-b	2012	China	Asian	Population	1919	1840	433	971	493	389	921	528	Taqman	matched	0.734
microRNA-499 rs3746444 G>A							GG	AG	AA	GG	AG	AA			
Sung JH	2016	Korea	Korean	Hospital	522	535	9	155	358	13	168	354	PCR-RFLP	matched	0.182
Xiong XD	2014	China	Asian	Hospital	295	283	3	65	227	4	67	212	PCR-RFLP	unmatched	0.616
Chen CR	2014	China	Asian	Hospital	919	889	70	237	612	37	246	606	PCR-LDR	unmatched	0.062
Chen L	2013	China	Asian	Hospital	658	658	46	149	463	26	158	474	Taqman	matched	0.007
Zhi H	2012	China	Asian	Hospital	916	584	86	201	629	21	167	396	PCR-RFLP	matched	0.517
Yang Y-a	2012	China	Asian	Population	853	948	28	210	589	28	212	683	Taqman	matched	0.023
microRNA-149 rs71428439 G>A							GG	AG	AA	GG	AG	AA			
Chen CR	2014	China	Asian	Hospital	919	889	155	389	375	124	381	384	PCR-LDR	unmatched	0.062
Ding SL	2013	China	Asian	NA	289	296	64	130	95	38	126	132	PCR-DNA sequencing	matched	0.360

CHD: coronary heart disease. HWE: Hardy-Weinberg equilibrium. ^a^ HWE in control. NA: not available

### Quantitative analysis

### Meta-analysis for microRNA-146a rs2910164 G>C polymorphism

**Figure 2:**
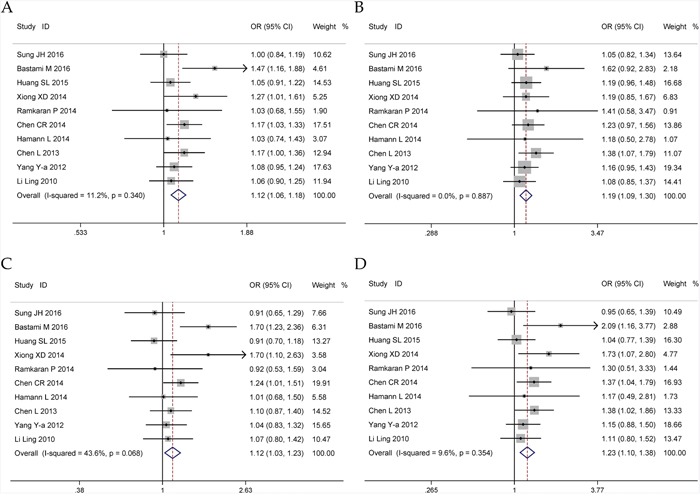
Forests for microRNA-146a rs2910164 G>C polymorphism and CHD **A**. allele model (C vs. G); **B**. recessive model (CC vs. GG + GC); **C**. dominant model (GC + CC vs. GG); **D**. homozygote model (CC vs. GG).

**Figure 3:**
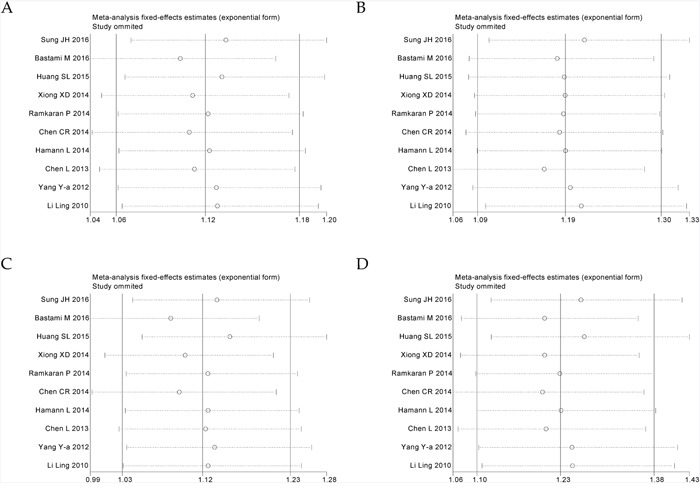
Sensitivity analyses for microRNA-146a rs2910164 G>C polymorphism and CHD **A**. allele model (C vs. G); **B**. recessive model (CC vs. GG + GC); **C**. dominant model (GC + CC vs. GG); **D**. homozygote model (CC vs. GG).

**Figure 4:**
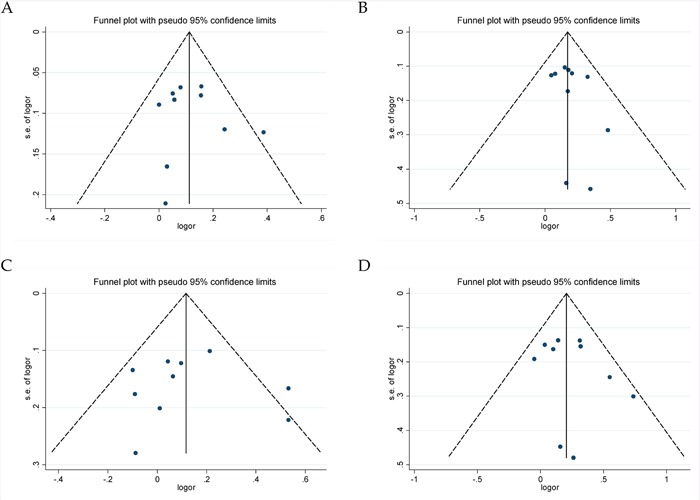
Funnel plots for microRNA-146a rs2910164 G>C polymorphism and CHD **A**. allele model (C vs. G); **B**. recessive model (CC vs. GG + GC); **C**. dominant model (GC + CC vs. GG); **D**. homozygote model (CC vs. GG).

Ten eligible studies including 4,996 cases and 5,644 controls were included to assess the association between miR-146a rs2910164 polymorphism and CHD risk. The heterogeneity in all genetic models was not significant statistically (*I^2^*<0.5). So we used the fixed effect model to calculate the ORs and 95% CIs. Overall, an increased CHD risk was detected in all five genetic models (C vs. G: OR = 1.12, 95% CI = 1.06–1.18, *P*<0.01, *I^2^* = 11.2%; CC vs. GG+GC: OR = 1.19, 95% CI = 1.09–1.30, *P*<0.01, *I^2^* = 0%; GC + CC vs. GG: OR = 1.12, 95% CI = 1.03–1.23, *P* = 0.012, *I^2^* = 43.6%; CC vs. GG: OR = 1.23, 95% CI = 1.10–1.38, *P*<0.01, *I^2^* = 9.6%; GC vs. GG: OR = 1.06, 95% CI = 0.97–1.17, *P* = 0.211, *I^2^* = 46.7%) (Figure 2, Table 2). Subgroup analyses of ethnicity disclosed similar results in Asians. In addition, significant associations were observed in subgroup analyses by source of controls and genotyping method (Table 2). The sensitivity analysis showed that the pooled ORs with corresponding 95%CI were not qualitatively changed by any single study in allelic, recessive, homozygous and heterozygous models, but dominant model (Figure 3). Publication bias was estimated by visual inspection of funnel plot and Egger's test, and the results revealed no asymmetrical evidence (Figure 4). The data of Egger's test supported the above results further (C vs. G: *P* = 0.682; CC vs. GG + GC: *P* = 0.283; GC + CC vs. GG: *P* = 0.911; CC vs. GG: *P* = 0.379; GC vs. GG: *P* = 0.877).

**Table 2:** Summary ORs and 95% CI of microRNA-146a rs2910164 polymorphisms and CHD risk

Locus	N^*^	Allele		Recessive		Dominant		Homozygote		Heterozygote	
		OR (95%CI) P	*I2(%)*	OR (95%CI) P	*I2(%)*	OR (95%CI) P	*I2(%)*	OR (95%CI) P	*I2(%)*	OR (95%CI) P	*I2(%)*
Total	10	1.12 (1.06-1.18) <0.01	11.2	1.19 (1.09-1.30) <0.01	0	1.12 (1.03-1.23) 0.012	43.6	1.23 (1.10-1.38) <0.01	9.6	1.06 (0.97-1.17) 0.211	46.7
Ethnicity											
Asian	8	1.10 (1.04-1.17) <0.01	0	1.18 (1.08-1.29) <0.01	0	1.09 (0.99-1.20) 0.083	22.6	1.20 (1.07-1.35) <0.01	0	1.03(0.93-1.14) 0.631	30.5
Caucasian	2	1.25 (0.88-1.77) 0.205	66.5	1.47 (0.92-2.35) 0.108	0	1.33 (0.80-2.21) 0.277	75.0	1.74 (1.07-2.84) 0.025	13.4	1.29 (0.79-2.11) 0.312	70.9
Source of controls											
Population	2	1.08 (0.95-1.22) 0.247	0	1.17 (0.96-1.42) 0.130	0	1.04 (0.85-1.27) 0.733	0	1.15 (0.89-1.49) 0.281	0	0.98 (0.80-1.22) 0.883	0
Hospital	6	1.11 (1.04-1.19) <0.01	0	1.18 (1.07-1.31) <0.01	0	1.11 (0.99-1.24) 0.067	40.9	1.22 (1.07-1.39) <0.01	23.8	1.04 (0.93-1.17) 0.470	47.0
Method
Taqman	4	1.15 (1.03-1.29) 0.017	50.0	1.24 (1.10-1.41) <0.01	0	1.14 (0.90-1.43) 0.281	67.3	1.23 (1.05-1.44) 0.012	41.9	1.05 (0.82-1.36) 0.698	69.9
PCR-RFLP	4	1.07 (0.97-1.19) 0.177	0	1.10 (0.94-1.28) 0.227	0	1.09 (0.91-1.31) 0.347	45.0	1.16 (0.94-1.43) 0.165	22.9	1.05 (0.87-1.28) 0.598	44.2
Age and sex matched	6	1.11 (1.03-1.19) <0.01	35.3	1.21 (1.08-1.35) <0.01	0	1.08 (0.90-1.29) 0.420	52.7	1.18 (1.02-1.37) 0.024	25.0	1.00 (0.83-1.22) 0.981	54.1
Controls in HWE	9	1.13 (1.06-1.20) <0.01	17.0	1.21 (1.10-1.33) <0.01	0	1.13 (1.03-1.25) 0.012	49.4	1.25 (1.11-1.41) <0.01	15.7	1.07 (0.92-1.25) 0.396	52.5

^*^ Numbers of comparisons. PCR-RFLP: polymerase chain reaction-based restriction fragment length polymorphism. HWE: Hardy-Weinberg equilibrium.

### Meta-analysis for microRNA-196a2 rs11614913 T>C polymorphism

**Figure 5:**
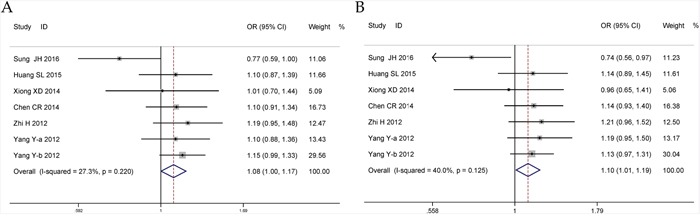
Forests for microRNA-196a2 rs11614913 T>C polymorphism and CHD **A**. dominant model (TC + CC vs. TT); **B**. heterozygote model (TC vs. TT).

Seven original studies involving 6,668 cases and 6,335 controls were analyzed for miRNA-196a2 rs11614913 T>C polymorphism and CHD susceptibility. In the overall analysis, significant associations were found in the dominant model (TC + CC vs. TT: OR = 1.08, 95%CI = 1.00–1.17, *P* = 0.046, *I^2^*= 27.3%) and heterozygous model (TC vs. TT: OR = 1.10, 95%CI = 1.01–1.19, *P* = 0.029, *I^2^*= 40%) (Figure 5, Table 3). In the stratified analysis, significant results were observed in group with population-based controls as well as genotyping method of Taqman (Table 3). Publication bias analyses were performed, and the shapes of funnel plots (Supplementary Figure 1) were consistent with the Egger's test approved (C vs. T: *P* = 0.262; CC vs. TT + TC: *P* = 0.650; TC + CC vs. TT: *P* = 0.226; CC vs. TT: *P* = 0.220; TC vs. TT: *P* = 0.292). However, when sensitivity analysis was performed, some changes of the pooled ORs were detected under both dominant and heterozygous models (Supplementary Figure 2).

**Table 3:** Summary ORs and 95% CI of microRNA-196a2 rs11614913 polymorphisms and CHD risk

Locus	N^*^	Allele		Recessive		Dominant		Homozygote		Heterozygote	
		OR (95%CI) P	*I2(%)*	OR (95%CI) P	*I2(%)*	OR (95%CI) P	*I2(%)*	OR (95%CI) P	*I2(%)*	OR (95%CI) P	*I2(%)*
Total	7	1.03 (0.98-1.09) 0.252	0	0.99 (0.90-1.08) 0.801	6.0	1.08 (1.00-1.17) 0.046	27.3	1.05 (0.95-1.16) 0.370	0	1.10 (1.01-1.19) 0.029	40.0
Source of controls											
Population	2	1.03 (0.91-1.17) 0.615	60.3	0.95 (0.69-1.30) 0.736	81.3	1.13 (1.00-1.28) 0.042	0	1.05 (0.80-1.39) 0.711	64.8	1.15 (1.01-1.30) 0.032	0
Hospital	5	1.02 (0.95-1.09) 0.671	0	0.98 (0.87-1.11) 0.793	0	1.05 (0.94-1.17) 0.389	44.8	1.01 (0.88-1.17) 0.863	0	1.04 (0.87-1.24) 0.666	55.4
Method											
Taqman	3	1.04 (0.97-1.11) 0.242	25.3	0.95 (0.78-1.16) 0.604	63.9	1.13 (1.01-1.25) 0.030	0	1.08 (0.94-1.23) 0.299	34.2	1.15 (1.02-1.28) 0.017	0
PCR-RFLP	3	1.01 (0.91-1.12) 0.847	30.4	1.04 (0.87-1.25) 0.661	0	0.98 (0.74-1.29) 0.867	68.5	1.02 (0.83-1.25) 0.874	0	0.95 (0.69-1.32) 0.775	72.4
Age and sex matched	5	1.03 (0.97-1.09) 0.315	30.4	0.99 (0.90-1.09) 0.825	28.9	1.07 (0.93-1.22) 0.342	50.4	1.05 (0.94-1.18) 0.387	18.0	1.08 (0.93-1.26) 0.316	57.3

^*^ Numbers of comparisons. PCR-RFLP: polymerase chain reaction-based restriction fragment length polymorphism.

### Meta-analysis for microRNA-499 rs3746444 A>G polymorphism

**Figure 6:**
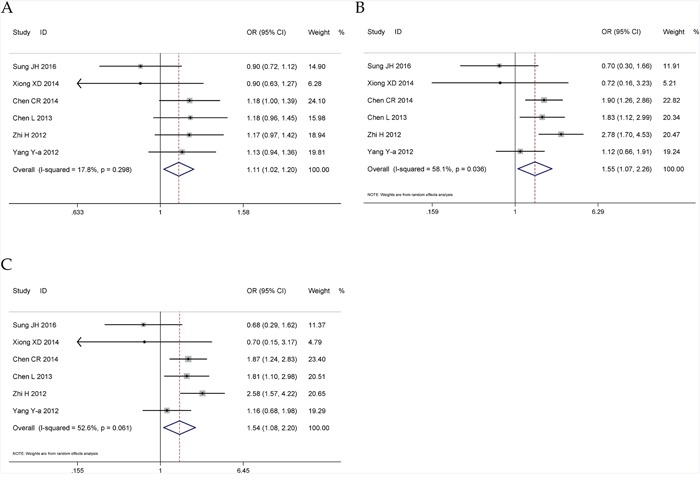
Forests for microRNA-499 rs3746444 A>G polymorphism and CHD **A**. allele model (G vs. A); **B**. recessive model (GG vs. AA + AG); **C**. homozygote model (GG vs. AA).

Six relevant studies comprising 4,163 patients and 3,897 controls were included in the meta-analysis for miRNA-499 rs3746444 A>G polymorphism and CHD risk. The pooled analyses indicated that this polymorphism was associated with an increased risk of CHD in three genetic models (G vs. A: OR = 1.11, 95% CI = 1.02–1.20, *P* = 0.015, *I^2^* = 17.8%; GG vs. AA + AG: OR = 1.55, 95% CI = 1.07–2.27, *P* = 0.022, *I^2^*= 58.1%; GG vs. AA: OR = 1.54, 95% CI = 1.08–2.20, *P* = 0.017, *I^2^*= 52.6%) (Figure 6, Table 4). Subsequent subgroup analyses revealed similar results in the hospital-based control group, genotyping method of Taqman group as well as age and sex matched group (Table 4). No significant publication bias was found, indicating that the meta-analysis results are reliable (G vs. A: *P* = 0.092; GG vs. AA + AG: *P* = 0.156; AG + GG vs. AA: *P* = 0.182; GG vs. AA: *P* = 0.198; AG vs. AA: *P* = 0.821) (Supplementary Figure 3). However, further sensitivity analysis revealed that omission of each study made some significant differences on the findings (Supplementary Figure 4).

**Table 4:** Summary ORs and 95% CI of microRNA-499 rs3746444 polymorphisms and CHD risk

Locus	N^*^	Allele		Recessive		Dominant		Homozygote		Heterozygote	
		OR (95%CI) P	*I2(%)*	OR (95%CI) P	*I2(%)*	OR (95%CI) P	*I2(%)*	OR (95%CI) P	*I2(%)*	OR (95%CI) P	*I2(%)*
Total	6	1.11 (1.02-1.20) 0.015	17.8	1.55 (1.07-2.27) 0.022	58.1	1.03 (0.94-1.13) 0.545	0	1.54 (1.08-2.20) 0.017	52.6	0.95 (0.85-1.05) 0.275	21.8
Source of controls											
Population	1	1.13 (0.94-1.36) 0.199	NA	1.12 (0.66-1.91) 0.676	NA	1.15 (0.93-1.42) 0.193	NA	1.16 (0.68-1.98) 0.588	NA	1.15 (0.92-1.43) 0.218	NA
Hospital	5	1.10 (1.01-1.21) 0.039	33.7	1.68 (1.11-2.55) 0.014	55.9	1.00 (0.90-1.12) 0.990	0	1.65 (1.10-2.46) 0.015	52.4	0.90 (0.80-1.01) 0.061	0
Method											
Taqman	2	1.15 (1.01-1.32) 0.042	0	1.46 (1.02-2.10) 0.038	42.7	1.12 (0.96-1.31) 0.156	0	1.48 (1.03-2.12) 0.034	30.1	1.07 (0.90-1.26) 0.446	0.8
PCR-RFLP	3	1.03 (0.90-1.17) 0.693	48.8	1.24 (0.42-3.66) 0.693	77.9	0.93 (0.79-1.08) 0.330	0	1.20 (0.42-3.39) 0.737	76.1	0.84 (0.71-0.99) 0.033	0
Age and sex matched	4	1.10 (1.00-1.22) 0.052	27.5	1.51 (0.89-2.57) 0.127	71.1	1.03 (0.92-1.15) 0.631	0	1.49 (0.91-2.45) 0.113	66.5	0.94 (0.79-1.12) 0.493	52.7
Controls in HWE	4	1.08 (0.98-1.20) 0.132	45.2	1.55 (0.86-2.79) 0.144	66.9	0.98 (0.87-1.11) 0.744	0	1.51 (0.85-2.66) 0.159	64.3	0.88 (0.77-1.00) 0.050	0

^*^ Numbers of comparisons. PCR-RFLP: polymerase chain reaction-based restriction fragment length polymorphism. HWE: Hardy-Weinberg equilibrium. NA: not available.

### Meta-analysis for microRNA-149 rs71428439 A>G polymorphism

A total of 2 studies with 1,208 cases and 1,185 controls were selected in the meta-analysis. This polymorphism was not found to be significantly associated with CHD risk in all five models (G vs. A: OR = 1.30, 95% CI = 0.94–1.79, *P* = 0.107, *I^2^* = 82.1%; GG vs. AA+AG: OR = 1.50, 95% CI = 0.98–2.27, *P* = 0.059, *I^2^* = 64.2%; AG + GG vs. AA: OR = 1.31, 95% CI = 0.89–1.93, *P* = 0.169, *I^2^* = 75.8%; GG vs. AA: OR = 1.67, 95% CI = 0.93–3.01, *P* = 0.086, *I^2^* = 78.1%; AG vs. AA: OR = 1.18, 95% CI = 0.87–1.59, *P* = 0.281, *I^2^* = 55.6%).

## DISCUSSION

Coronary heart disease is the most common cause of morbidity and mortality in most regions worldwide. Although we have conducted some major advances in the understanding of cardiovascular disease in more recent decades, detailed pathogenesis of CHD remain to be explored. Nowadays, the association between polymorphisms of microRNAs and CHD risk is drawing more and more attention.

In the current meta-analysis, we comprehensively investigated the associations between microRNA-146a rs2910164 G>C, microRNA-196a2 rs11614913 T>C, microRNA-499 rs3746444 A>G and microRNA-149 rs71428439 A>G polymorphisms and CHD risk according to thirteen included case-control studies, consisting of 8,120 patients and 8,364 controls. Overall, significant increased risks of CHD were observed for microRNA-146a rs2910164, microRNA-196a2 rs11614913 and microRNA-499 rs3746444, but not miRNA-149 rs71428439.

As for miRNA-146a rs2910164 G>C, this is the latest and largest meta-analysis investigated the association with CHD risk. Compared with the previous meta-analysis with four studies including 2506 subjects [35], we found that the significant association existed in recessive model, as well as no association in heterozygous model. The advantages of our analysis are as follows. First, our meta-analysis had much larger sample size: we added another six recent studies involving 8,134 subjects which were not part of the previous meta-analysis [23, 24, 27, 29, 31, 32]. Second, sensitivity analyses showed that our results were statistically robust in four genetic models. Also, no significant publication bias was detected in our meta-analysis. Third, we performed a more comprehensive subgroup analyses. Stratification by ethnicity showed an increased CHD risk for microRNA-146a rs2910164 G>C polymorphism in Asians. Furthermore, similar increased results were observed in the group with genotyping method of Taqman, rather than PCR-RFLP. It revealed that Taqman was a more useful genotyping method to improve the accuracy of experiment.

To the best of our knowledge, this is the first meta-analysis assessing the association of miRNA-196a2 rs11614913 T>C, miRNA-499 rs3746444 A>G, and miRNA-149 rs71428439 A>G polymorphisms with CHD susceptibility. Interestingly, by increasing the sample size, the results of the combined analysis revealed a significant association with CHD risk for microRNA-196a2 rs11614913, even though no association was found in each single original study. How can we explain the association of miRNA-196a2 rs11614913 with CHD susceptibility? First, the miRNA-196a2 rs11614913 polymorphism involved a T to C nucleotide substitution and situated in the 3p strand of mature miRNA regions, which might affect both pre-miRNA maturation of 5p and 3p miRNAs and the interacting of target mRNAs to 3p mature miRNAs [36]. Second, it has been reported that miR-196a2 was closely associated with the regulation of annexin A1 (ANXA1) [37]. As an important modulator in atherosclerosis, ANXA1 can inhibit not only the monocyte adhesion to endothelium but also the expression of inflammatory enzymes, such as inducible cyclooxygenase 2 (COX-2) and phospholipase A2 [38, 39]. Additionally, the predicted targets of miR-196a2 included hundreds of genes ( http://www.targetscan.org). There also existed the possibility that other targets of miR-196a2 might play some roles in the development of CHD, despite it was unknown by far.

Our meta-analysis had several limitations. First of all, the ethnicity of most subjects was Asian in the current study and this restricted the general application of the results to other populations. Second, only articles published in English or Chinese were selected, potentially causing a language bias. Third, in the sensitivity analysis for miRNA-196a2 rs11614913 T>C and miRNA-499 rs3746444 A>G, we found that omission of each study made some significant differences on the results. Although it may be explained by the small number of studies included, the caution should be indicated when interpreting the association of these two miRNA polymorphisms with CHD. Third, the heterogeneity existed in our meta-analysis for miRNA-499 rs3746444 A>G and microRNA-149 rs71428439 A>G. For rs3746444, although subgroup and sensitivity analyses were performed, unfortunately, we have not found the sources of heterogeneity. Also, as for rs71428439, only two included studies were too small to analyze the sources of heterogeneity. Fourth, CHD is both multi-factorial disease influenced by genetic and environmental factors. However, in our current meta-analysis, the inter-gene and gene-environment interactions could not be conducted owing to the data deficiency. Last but not the least, genetic epidemiological studies show different genetic variants can predispose to different subtypes of CHD [40–42]. So subtypes of CHD, such as myocardial infarction, acute coronary syndrome and stable angina should be further analyzed. Unfortunately, we could not assess the difference among these subtypes of CHD due to insufficient statistical data in the literature.

In conclusion, the current meta-analysis demonstrated that three functional polymorphisms of microRNA-146a rs2910164 G>C, microRNA-196a2 rs11614913 T>C and microRNA-499 rs3746444 A>G might take important part in the development of CHD. Considering the limitations in the current meta-analysis, our results should be interpreted with caution. More eligible studies with rigorous design are needed to confirm the association of above polymorphisms in miRNA and CHD risk in the future.

## MATERIALS AND METHODS

### Search strategy

We searched four electronic databases (Pubmed, Embase, CNKI and Wanfang) for articles written in English or Chinese published prior to August 31, 2016. The following medical subject heading terms were used: (microRNA OR miRNA) AND (myocardial infarction OR ischemic heart disease OR ischaemic heart disease OR coronary heart disease OR coronary artery disease OR coronary syndrome OR coronary stenosis OR coronary disease OR cardiovascular disease OR CAD OR CHD OR MI) AND (genotype OR gene OR allele OR polymorphism OR variant OR SNP).

### Study selection

All selected studies had to meet the following criteria: (1) published studies based on case-control design assessing the association of rs2910164 G>C in miR-146a, rs11614913 T>C in miR-196a2, rs3746444 A>G in miR-499 and rs71428439 A>G in miR-149 with CHD risk; (2) availability of allele or genotype frequency for calculating odds radio (OR) and their 95% confidence interval (CI). Studies were excluded if they investigated the progression, severity, phenotype modification, response to treatment, survival or family based studies. Moreover, meeting abstracts, case reports, editorials, review articles and non-English and non-Chinese articles were also excluded. For duplicate publications, the one with more complete design or larger sample size was finally selected.

### Data extraction

The two of the authors independently extracted the data from each relevant study including the first author, publication year, study country/region, ethnicity of participants (such as Asian or Caucasian), sources of controls, genotyping method, case-control matched status, HWE status of controls and number of genotypes in CHD cases and controls. Disagreements were reconciled through group discussion. The Hardy-Weinberg equilibrium (HWE) was calculated based on the genotypes of the controls.

### Statistical analysis

Heterogeneity among studies was examined with the *I^2^* statistic and *I^2^*>50% indicates significant heterogeneity between the studies. Based on the test of heterogeneity, a pooled OR was calculated by using fixed or random effect model, along with the 95% CI to measure the strength of the genetic association. For the microRNA-146a rs2910164 G>C polymorphism, the pooled ORs were obtained for the allele contrast (C vs. G), recessive model (CC vs. GG+GC), dominant model (GC+CC vs. GG), homozygous (co-dominant) model (CC vs. GG) and heterozygous (co-dominant) model (GC vs. GG). Similar genetic models were also assessed for microRNA-196a2 rs11614913 T>C, microRNA-499 rs3746444 A>G and microRNA-149 rs71428439 A>G variants. Subgroup analyses of ethnicity, source of controls, genotyping methods, case-control matched status and HWE status of controls were also submitted to statistical testing. In order to evaluate the stability of the results, sensitivity analysis was used, which meant omitting one study at a time, and then compared to show whether a significant difference existed between the former and the latter results. Publication bias was examined by the visual inspection of funnel plot, and Egger's regression test. Data were analyzed and processed using Stata 12.0 (Stata Corporation, College Station, TX, USA). *P*<0.05 was considered statistically significant.

## SUPPLEMENTARY MATERIALS FIGURES AND TABLES


