# Supplementary material for: Significant association between functional microRNA polymorphisms and coronary heart disease susceptibility: a comprehensive meta-analysis involving 16484 subjects
Source: Oncotarget. 2016 Dec 27;8(4):5692–702. doi: 10.18632/oncotarget.14249 (PMC5351582; doi:10.18632/oncotarget.14249)
Supplement: Supplementary file 1 [file oncotarget-08-5692-s001.pdf]

Significant association between functional microRNA polymorphisms and coronary heart disease susceptibility: a comprehensive meta-analysis involving 16484 subjects

SUPPLEMENTARY FIGURES

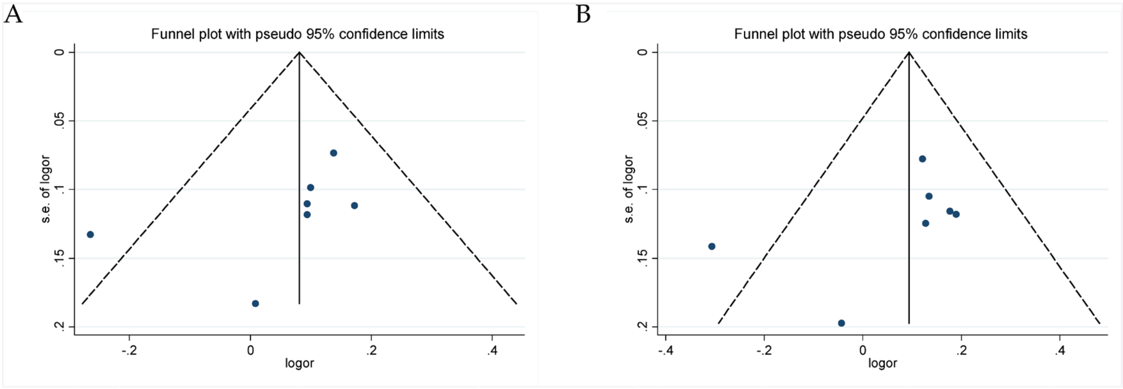

Supplementary Figure 1: Funnel plots for microRNA-196a2 rs11614913 T>C polymorphism and CHD. A. dominant model (TC + CC vs. TT); **B.** heterozygote model (TC vs. TT).

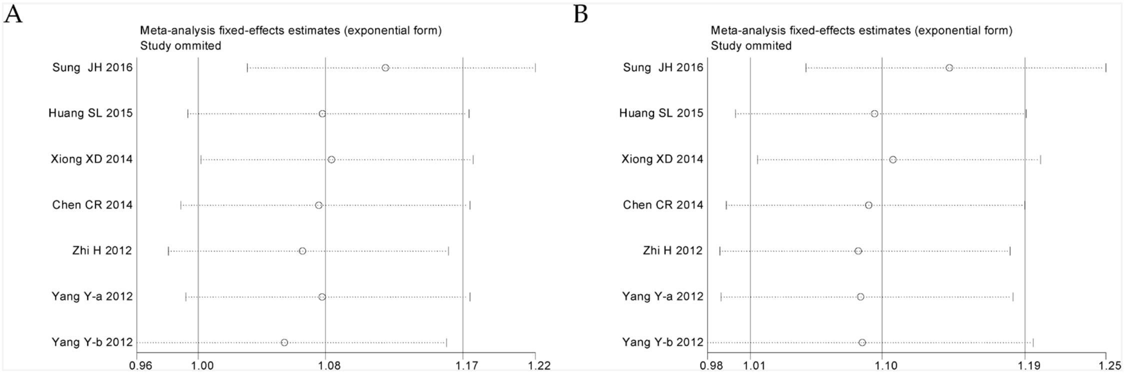

Supplementary Figure 2: Sensitivity analyses for microRNA-196a2 rs11614913 T>C polymorphism and CHD. A. dominant model (TC + CC vs. TT); **B.** heterozygote model (TC vs. TT).

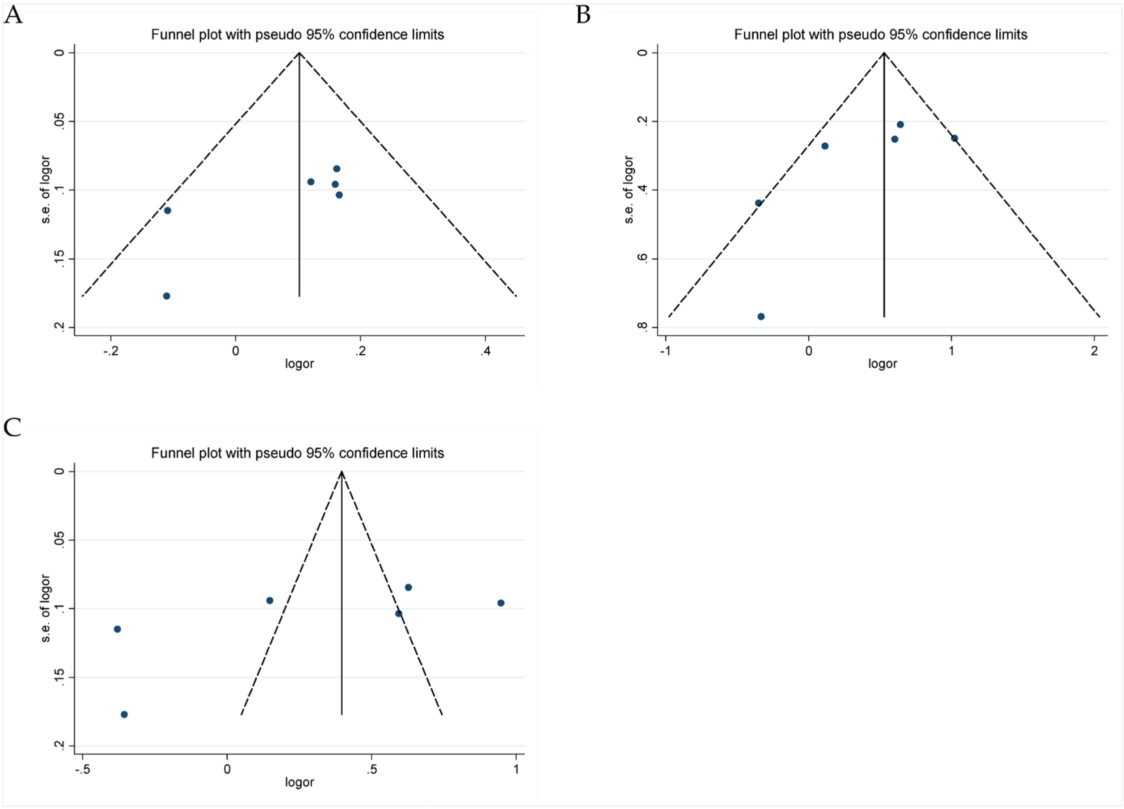

Supplementary Figure 3: Funnel plots for microRNA-499 rs3746444 A>G polymorphism and CHD. A. allele model (G vs. A); **B.** recessive model (GG vs. AA + AG); **C**. homozygote model (GG vs. AA).

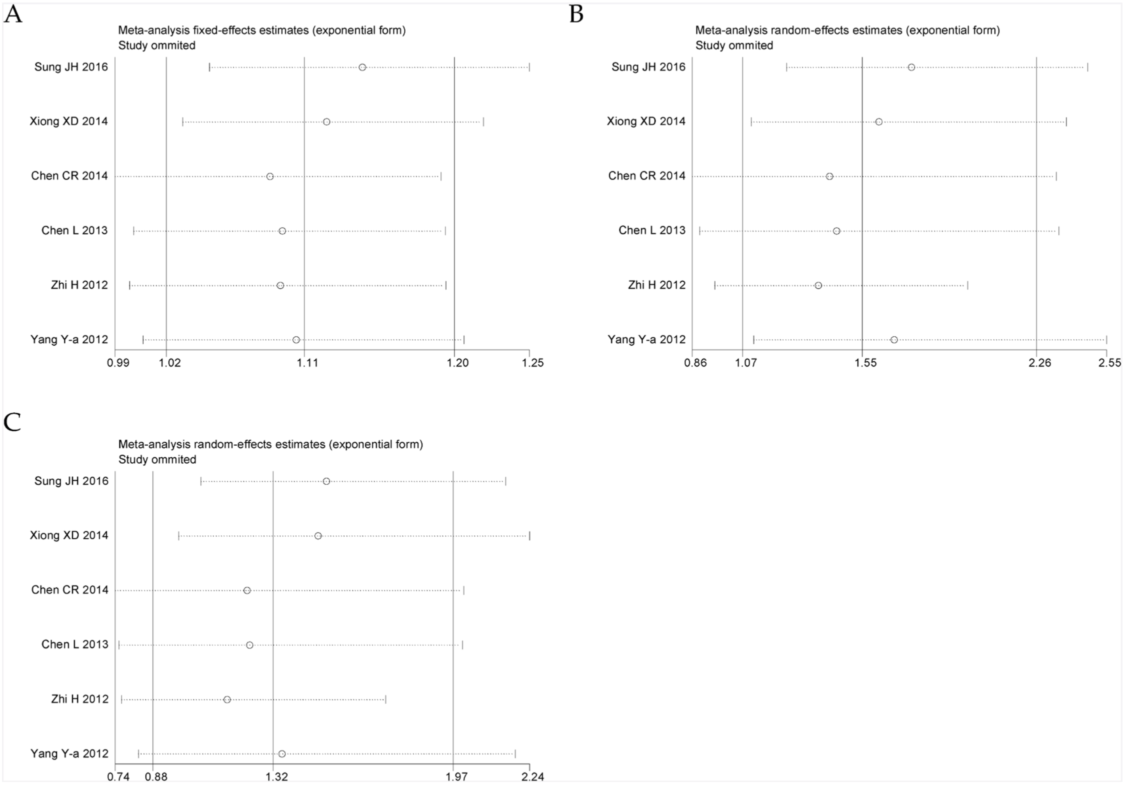

Supplementary Figure 4: Sensitivity analyses for microRNA-499 rs3746444 A>G polymorphism and CHD. A. allele model (G vs. A); **B.** recessive model (GG vs. AA + AG); **C.** homozygote model (GG vs. AA).
